# Inhaled sphingosine has no adverse side effects in isolated ventilated and perfused pig lungs

**DOI:** 10.1038/s41598-021-97708-3

**Published:** 2021-09-20

**Authors:** Henning Carstens, Katharina Kalka, Rabea Verhaegh, Fabian Schumacher, Matthias Soddemann, Barbara Wilker, Simone Keitsch, Carolin Sehl, Burkhard Kleuser, Thorsten Wahlers, Gerald Reiner, Achim Koch, Ursula Rauen, Erich Gulbins, Markus Kamler

**Affiliations:** 1 Department of Thoracic and Cardiovascular Surgery, University Hospital Essen, University of Duisburg-Essen, Hufelandstrasse 55, 45122 Essen, Germany; 2 Institute of Molecular Biology, University of Duisburg-Essen, Hufelandstrasse 55, 45122 Essen, Germany; 3 Department of Toxicology, University of Potsdam, Arthur-Scheunert-Allee 114-116, 14558 Nuthetal, Germany; 4 Department of Surgery, University of Cincinnati, Medical School, 231 Albert Sabin Way, ML0558, Cincinnati, OH 45267 USA; 5 Institute of Pharmacy, Freie Universität Berlin, Königin-Luise-Str. 2+4, 14195 Berlin, Germany; 6 Department of Cardiothoracic Surgery, Heart Center, University of Cologne, Kerpener Strasse 61, 50924 Cologne, Germany; 7 Cardiac Surgery for Congenital Heart Disease, University Medical Center Hamburg- Eppendorf, Martinistrasse 52, 20251 Hamburg, Germany; 8 Department of Veterinary Clinical Sciences, Swine Clinic, Justus-Liebig-University, Giessen, Germany

**Keywords:** Drug safety, Drug delivery, Infectious diseases

## Abstract

*Ex-vivo* lung perfusion (EVLP) systems like XVIVO are more and more common in the setting of lung transplantation, since marginal donor-lungs can easily be subjected to a performance test or be treated with corticosteroids or antibiotics in high dose regimes. Donor lungs are frequently positive in bronchoalveolar lavage (BAL) bacterial cultures (46–89%) which leads to a donor-to-recipient transmission and after a higher risk of lung infection with reduced posttransplant outcome. We have previously shown that sphingosine very efficiently kills a variety of pathogens, including *Pseudomonas aeruginosa*, *Staphylococcus aureus* and *epidermidis*, *Escherichia coli* or *Haemophilus influenzae*. Thus, sphingosine could be a new treatment option with broadspectrum antiinfective potential, which may improve outcome after lung transplantation when administered prior to lung re-implantation. Here, we tested whether sphingosine has any adverse effects in the respiratory tract when applied into isolated ventilated and perfused lungs. A 4-h EVLP run using minipig lungs was performed. Functional parameters as well as perfusate measurements where obtained. Biopsies were obtained 30 min and 150 min after inhalation of sphingosine. Tissue samples were fixed in paraformaldehyde, embedded in paraffin and sectioned. Hemalaun, TUNEL as well as stainings with Cy3-coupled anti-sphingosine or anti-ceramide antibodies were implemented. We demonstrate that tube-inhalation of sphingosine into *ex-vivo* perfused and ventilated minipig lungs results in increased levels of sphingosine in the luminal membrane of bronchi and the trachea without morphological side effects up to very high doses of sphingosine. Sphingosine also did not affect functional lung performance. In summary, the inhalation of sphingosine results in an increase of sphingosine concentrations in the luminal plasma membrane of tracheal and bronchial epithelial cells. The inhalation has no local side effects in *ex-vivo* perfused and ventilated minipig lungs.

## Introduction

One of the most important upcoming challenges in global health is the prevention and treatment of bacterial infections with accelerating antibiotic resistances. Bacterial pneumonia and in particular ventilator-associated pneumonia (VAP) have high mortality rates, which are ranging from 24% up to 76% in specific settings^1^. The most common pathogen worldwide in community-acquired pneumonia (CAP) (17% of all cases), hospital-acquired pneumonia (HAP) (25% of all cases)^2^, and ventilator-associated pneumonia in intensive care units (28% of all cases)^3^ is *Pseudomonas aeruginosa* (PA). Next to the acute pulmonary infections, PA is also common among chronic pulmonary infections in patients suffering from chronic obstructive pulmonary disease (COPD) and bronchiectasis^4^ or cystic fibrosis (CF)^5^. There is an increase of multidrug-resistant (MDR) bacterial pathogens, such as PA, Acinetobacter species, and methicillin-resistant *Staphylococcus aureus* (SA), recorded for HAP and VAP over the last years^6^. Due to this growing global public health issue, national governments addressed this growing threat and set priorities for the quest of novel antimicrobial agents^7^. Therefore, we investigated pulmonary effects of sphingosine (SPH), which was recently identified as a lipid with marked antimicrobial potency. Sphingosine is a sphingoid long-chain base which is formed by catabolic degradation from ceramide by ceramidases. Sphingosine kills several bacterial species, for instance PA, *Staphylococus aureus* (SA) (even methicillin resistant SA; MRSA)*, Acinetobacter baumannii*, *Escherichia coli*, and *Neisseria meningitides *in vitro and in vivo^8–13^. Previous studies have demonstrated that sphingosine is abundantly expressed on the luminal side of nasal to bronchial epithelial cells in wild-type mice, while sphingosine is greatly reduced in epithelial cells of cystic fibrosis (CF) patients and mice, due to reduced activity of the acid ceramidase in CF epithelial cells^8,9,12^. A return to normal sphingosine levels in airways upon inhalation of sphingosine also reduced the susceptibility of CF mice to develop pulmonary infections indicating that sphingosine acts as a natural antibacterial agent in the airways^8^. We investigated whether administration of sphingosine to *ex-vivo* perfused and ventilated minipig-lungs (EVLP) via nebulization had no side effects in epithelial cells of the respiratory tract. In addition, EVLP systems like XVIVO are more and more common in the setting of lung transplantation, where marginal donor-lungs can easily be subjected to a performance test or be treated with corticosteroids or antibiotics in high dose regimes^14,15^. Donor lungs are frequently positive in bronchoalveolar lavage (BAL) bacterial cultures (46–89%) which leads to a donor-to-recipient transmission and subsequently to a higher risk of lung infection with reduced posttransplant outcome^6,16,17^. Thus, inhalative sphingosine treatment prior to lung re-implantation may reduce bacterial counts and may lead to an improved outcome after lung transplantation if it can be inhaled.

## Materials and methods

### Animals

For the organ procurement one year old Goettingen mini pigs (Ellegaard, Soroe Landevej 302, 4261 Dalmose, Denmark) were used with supervison of Central Animal Laboratory of the University Duisburg-Essen accordingly to the “Principles of Laboratory and Animal care”^18^ with at least 10 days of quarantine. Institutional committee has approved the experiments, including any relevant details. All animals were checked by general examination for signs of respiratory diseases prior to the experiment. Additionally, samples of every experimental lung were taken and checked for typical porcine diseases which can affect lung functional outcome by real time PCR analysis. Ten 10 pigs were euthanized for organ procurements to achieve a group size of n = 3 for each of the three groups, one animal was excluded due to multiple positive results of porcine diseases.

Prior to euthanasia no intervention or medical application was conducted. Organ procurement was reported to the local authorities (Landesamt für Natur, Umwelt und Verbraucherschutz NRW) according to applicable german law (§ 1 VTMVO). We confirm that all experiments were performed in accordance with the relevant guidelines (including the ARRIVE guidelines) and regulations.

### Lung procurement

After ketamine (30 mg/kg, i.m.) and xylazine (2 mg/kg, i.m.) sedation pigs were anesthetized with midazolam (0.1 mg/kg, i.v.) and ketamine (0.3 mg/kg, i.v.) before euthanasia with potassium chloride (7,45%, 1.67 mL/kg, i.v.) was induced. After confirmed death (Maastricht Classification^19^), sternotomy was performed as previously described^20,21^. Lungs were flushed antegrade with 2 L of 4 °C Perfadex-Plus (XVIVO Perfusion, Gotheburg, Sweden) followed by a 2-h period of cold static preservation (CSP).

### *Ex-Vivo* lung perfusion (EVLP)

A 4-h EVLP run employing the XVIVO System (XVIVO Perfusion, Gotheburg, Sweden) following a modified Toronto protocol was implemented^20^. A pressure-controlled ventilation form was used to prevent barotrauma especially when ventilation was started and minipig lungs were distinct atelectatic^22^. The XVIVO System was built up with a mechanical ventilator (Hamilton-C2; Hamilton Medical AG, Bonaduz, Switzerland) and a centrifugal pump (Rotaflow; Maquet Cardio-pulmonary AG, Hirrlingen, Germany), which circulated perfusate through the system. A gas mixture of 6% O_2_, 8% CO_2_, and 86% N_2_ (named CRYSTAL gas, Air Liquide, Duisburg, Germany) was administered while the perfusate passed through a Quadrox PLS membrane oxygenator (Maquet Cardiopulmonary AG) to achieve deoxygenation before the perfusate was recirculated through the pulmonary artery. Fluids were warmed to 36 °C using a heat exchanger (HU 35, Maquet Cardiopulmonary AG). An acellular perfusate was utilized for the circuit containing modified Custodiol-N (Dr. Franz Köhler Chemie GmbH, Bensheim, Germany), 200 mL 10% human low-sodium albumin (CSL Behring GmbH, Hattersheim, Germany) and 35 mL 5% glucose (G5, B.Braun, Melsungen, Germany).

### Functional measurements

Pulmonary function parameters containing oxygenation capacity (ΔpO_2_), pulmonary vascular resistance (PVR), and the static / dynamic compliance (Cstat/Cdyn).

### Perfusate measurements

Perfusate analysis were performed hourly. Lactate levels as by-product of anaerobic cellular metabolism, activities of lactate dehydrogenase (LDH) as a marker of cell damage and alkaline phosphatase (AP) activity as a marker of pneumocyte type II injury were measured.

### Wet/dry-ratio

For the wet/dry-ratio small biopsies out of the lobus caudalis were collected prior to perfusion start and after the EVLP run to quantify the water content of lung tissue. Wet weight was measured immediately after removal and for the dry weight tissue samples underwent a 24-h desication at 65 °C.

### Inhalations

After balanced randomization inhalation groups were built (5 ml; 125 µM (n = 3), 500 µM (n = 3) of sphingosine and control with the solvent 10% octylglucopyranoside (OGP) (n = 3). Solubilization of sphingosine was achieved by dissolving sphingosine powder in OGP. One hour after organ perfusion was started a 15-min inhalation (Aerogen, Aerogen Solo, Galway, Ireland; particle size 3.54 µm) was implemented.

### Biopsies

Proximal bronchi biopsies were obtained 30 min and 150 min after inhalation end using a fiberoptic videoscope (Ambu A/S, Baltorpbakken 13, DK-2750 Ballerup, Denmark). In none of the lungs pathological changes or signs for tumors were observed. Tissue samples from larger bronchi were taken for histological and biochemical studies using toothed (alligator) forceps and underwent immediately fixing in 4% paraformaldehyde (PFA) for 40 h or shock-freezing in liquid nitrogen.

### Quantification of sphingosine and ceramides by HPLC–MS/MS

Shock-frozen tissue samples were subjected to lipid extraction using 1.5 mL methanol/chloroform (2:1, *v:v*) as described^23^. Extraction solvents contained d_7_-sphingosine, 17:0 ceramide and 16:0-d_31_-sphingomyelin (all Avanti Polar Lipids, Alabaster, USA) as internal standards. Chromatographic separations were achieved on a 1260 Infinity HPLC (Agilent Technologies, Waldbronn, Germany) equipped with a Poroshell 120 EC-C8 column (3.0 × 150 mm, 2.7 µm; Agilent Technologies). MS/MS analyses were carried out using a 6490 triple-quadrupole mass spectrometer (Agilent Technologies) operating in the positive electrospray ionization mode (ESI +). SPH and six sub-species (16:0, 18:0, 20:0, 22:0, 24:0 and 24:1) each of ceramides (Cer) and sphingomyelins (SM) were analyzed by selected reaction monitoring (SRM) as described^24^. Briefly, ceramides were monitored using the mass transition [M-H_2_O + H]^+^  → *m/z* 264.3, while [M + H]^+^  → *m/z* 184.1 was used for detection of sphingomyelins. Sphingosine and its deuterated internal standard were analyzed by the transitions *m/z* 300.3 → 282.3 (SPH) and *m/z* 307.3 → 289.3 (d_7_-SPH), respectively. Peak areas of Cer and SM sub-species, as determined with MassHunter software (Agilent Technologies), were normalized to those of their respective internal standards (17:0 Cer or 16:0-d_31_-SM) followed by external calibration in the range of 1 to 50 pmol on column. SPH was directly quantified via its stable-isotopically labeled internal standard d_7_-SPH (0.25 pmol on column). Quantification was performed with MassHunter Software (Agilent Technologies). Sphingosine and ceramide contents were normalized to total sphingomyelin (sum of six sub-species) and expressed as fmol / pmol total SM.

### Antibodies and reagents

Ceramide was stained in the histologies using the monoclonal mouse anti-ceramide antibody clone S58-9 (#MAB_0011, Glycobiotech). Sphingosine was detected by monoclonal mouse anti-sphingosine antibodies, clone NHSPH (#ALF-274042010, Alfresa Pharma Corporation). Cy3 donkey anti-mouse IgM F(ab)_2_ fragments (#715–166-020; Jackson ImmunoResearch) or Cy5-coupled donkey anti-mouse IgM antibody (#715–176-020; Jackson ImmunoResearch) were used as secondary antibodies for visualization.

### ImmunohistochemistryImmunohistochemistry

Stainings were performed as previously described^8,9,25–27^. Samples were fixed in 4% phosphate-buffered (PBS) paraformaldehyde (pH 7.2—7.4) for 48 h, washed and stepwise dehydrated with an ethanol to xylol gradient. Lung tissue biopsies were then embedded in paraplast and sectioned at 7 μm. Sections were dewaxed, rehydrated and antigens were retrieved by 30 min treatment with pepsin (Digest All; #003,009, Invitrogen) at 37 °C. Sections were washed with water and PBS (pH 7.4), unspecific binding sites were blocked by incubation in PBS supplemented with 0.05% Tween 20 (Sigma) and 5% fetal calf serum for 10 min. Samples were stained with anti-ceramide antibodies (1:100 dilution) or anti-sphingosine antibodies (1:1000 dilution) in H/S (132 mM NaCl, 20 mM HEPES [pH 7.4], 5 mM KCl, 1 mM CaCl_2_, 0.7 mM MgCl_2_, 0.8 mM MgSO_4_) plus 1% FCS at room temperature for 45 min. Sections were washed 3-times with PBS supplemented with 0.05% Tween 20, once with PBS and stained with Cy3-coupled anti-mouse IgM F(ab)_2_ fragments diluted 1:200 in H/S, 1% FCS for 30 min. Samples were washed as above and embedded in Mowiol. Sections were analyzed on a Leica TCS-SP5 confocal microscope employing a 40 × lens. Image analysis was performed using a Leica LCS software version 2.61 (Leica Microsystems, Mannheim, Germany) with identical settings for all samples.

### TUNEL assays

As previously described^28^, PFA-fixed samples were processed and sectioned as described above. Sections were microwaved with 0.1 M sodium citrate (pH 6.0) at 450 W for 5 min, washed twice in PBS and the TUNEL reaction was performed with 5 μL TUNEL enzyme, 20 μL TMR label and 25 μL TUNEL dilution per sample according to the instructions of the vendor (Roche). Samples were incubated for 60 min at 37 °C and washed 3-times in PBS. Finally, samples were incubated for 10 min at 70 °C in PBS, washed once in PBS and embedded in Mowiol in order to reduce background staining.

### Hemalaun stainings

As previously described^28^, paraffin sections of lung tissues were dewaxed, rehydrated and washed as described above followed by a 5 min staining with hemalaun. Sections were embedded in Mowiol and analyzed on a Leica TCS-SP5 confocal microscope employing a 40 × lens. Hemalaun stainings were scored as following: Grade 0: no change of the epithelial cell layer, basal membrane intact, no evidence of leukocyte influx, less than 2% pycnotic, i.e. dead cells. Grade 1: small disruptions of the epithelial cell layer, basal membrane intact, very minor leukocyte influx with few singular cells in the epithelial cell layer, less than 5% pycnotic, i.e. dead cells. Grade 2: Larger disruptions of the epithelial cell layer, basal membrane still intact, scattered leukocyte influx, less than 10% pycnotic, i.e. dead cells. Grade 3: Larger disruptions of the epithelial cell layer, disrupted basal membrane, massive leukocyte influx, more than 10% pycnotic, i.e. dead cells.

### Statistics

Three groups were built based on inhalation solution and concentration (sphingosine 125 µM (125 µM SPH); n = 3), sphingosine 500 µM (500 µM SPH); n = 3) and the solvent octylglucopyranoside (OGP; n = 3)). Data was explored in mean value (mean) and standard deviation (sd). Differences were considered significant at the level of *p* < 0.05 = *, *p* < 0.01 = ** and *p* < 0.001 = ***, Analysis of Variance (ANOVA) was used to test differences in means of the three independent groups. If normal distribution of these variables was rejected Kruskal–Wallis testing was applied. To reduce the familywise error rate in multiple comparison testing (post-hoc test) a Bonferroni correction was implemented. Statistical analysis was performed using SPSS Statistics 22 (IBM, Armonk, New York, US).

### Ethical statement

All animal experiments conform to internationally accepted standards and have been approved by the appropriate institutional review body, i.e. LANUV, Recklinghausen, Germany.

## Results

To test possible adverse effects of sphingosine in EVLP mini pig lungs, we measured oxygen capacity (∆pO_2_), static (Cstat) and dynamic compliance (Cdyn), pulmonary vascular resistance (PVR) after inhalation of a 5 ml suspension containing 125 µM, 500 µM sphingosine or OGP, the solvent of sphingosine. The results showed that sphingosine did not significantly affect functional performance during a 4-h EVLP run (Table 1). Water content of lung tissues sample increased in all groups during EVLP run without significant differences. Perfusate measurements revealed an increase of lactate levels and activity of LDH and AP. Statistical computing at 1, 2, 3 and 4 h did not yield significant differences (Table 1).

**Table 1:** Statistical analysis of the functional parameters ∆pO_2_ (difference between pulmonary arterial to pulmonary venous oxygen partial pressure in the perfusate), Cstat and Cdyn (static and dynamic lung compliance), PVR (pulmonary vascular resistance), as well as the ratio between wet and dry weight after EVLP run (wet/dry ration, W/D-ratio) and perfusate measurements of lactate levels, activity of lactate dehydrogenase (LDH) and alkaline phosphatase (AP) after inhalation of a 125 µM (SPH 125), 500 µM (SPH 500) and 10% OGP (OGP) suspension in a 4 h EVLP run.

Parameters	EVLP time	OGP		SPH 125 µM		SPH 500 µM		*p*-value
		n	Mean ± sd	n	Mean ± sd	n	Mean ± sd	
**Functional**								
ΔpO_2_	1 h	3	386 ± 17	3	291 ± 167	3	320 ± 92	*n.s*
	2 h	3	416 ± 92	3	295 ± 214	3	298 ± 97	*n.s*
	3 h	2	371 ± 25	3	285 ± 183	3	278 ± 75	*n.s*
	4 h	3	386 ± 105	3	250 ± 135	2	273 ± 173	*n.s*
	*p*-value		*n.s*		*n.s*		*n.s*	
PVR	1 h	2	530 ± 434	3	601 ± 772	3	223 ± 93	*n.s*
	2 h	2	466 ± 426	3	493 ± 579	3	252 ± 166	*n.s*
	3 h	2	651 ± 707	3	667 ± 576	3	310 ± 195	*n.s*
	4 h	2	612 ± 747	3	742 ± 645	2	431 ± 531	*n.s*
	*p*-value		*n.s*		*n.s*		*n.s*	
Cdyn	1 h	3	27 ± 8	3	17 ± 2	3	20 ± 9	*n.s*
	2 h	3	24 ± 9	3	16 ± 6	3	18 ± 8	*n.s*
	3 h	3	17 ± 4	3	15 ± 8	3	20 ± 4	*n.s*
	4 h	3	20 ± 17	3	12 ± 9	2	14 ± 12	*n.s*
	*p*-value		*n.s*		*n.s*		*n.s*	
Cstat	1 h	3	39 ± 22	3	19 ± 1	3	22 ± 10	*n.s*
	2 h	3	31 ± 20	3	18 ± 6	3	21 ± 8	*n.s*
	3 h	3	32 ± 27	3	17 ± 6	3	37 ± 24	*n.s*
	4 h	3	32 ± 36	3	13 ± 9	2	21 ± 10	*n.s*
	*p*-value		*n.s*		*n.s*		*n.s*	
W/D Ratio		3	88.41 ± 2.3	3	86.98 ± 3.2	3	87.57 ± 2.3	*n.s*
**Perfusate**								
Lactate	1 h	3	1.9 ± 0.42	3	1.2 ± 0.06	3	1.4 ± 0.45	*n.s*
	2 h	3	2.0 ± 0.49	3	1.9 ± 0.21	3	2.4 ± 0.35	*n.s*
	3 h	3	2.6 ± 0.72	3	2.2 ± 0.15	3	3.0 ± 0.27	*n.s*
	4 h	2	3.1 ± 0.85	3	2.7 ± 0.15	3	3.4 ± 0.20	*n.s*
	*p*-value		*n.s*		*n.s*		*n.s*	
LDH	Start	3	249 ± 151	3	200 ± 93	3	192 ± 164	*n.s*
	End	3	326 ± 195	3	296 ± 127	3	313 ± 158	*n.s*
AP	Start	3	7 ± 5	3	1 ± 2	3	3 ± 3	*n.s*
	End	3	21 ± 25	3	2 ± 3	3	5 ± 5	*n.s*
	*p*-value		*n.s*		*n.s*		*n.s*	

Due to technical issues in terms of PGM failure with missing log-files, some functional parameters only have two evaluable samples. Given is the mean ± SD, ANOVA.

Mass spectrometry (MS) analysis of lung tissues after EVLP run revealed a trend to a dose-dependent increase of bronchial sphingosine concentrations after inhalation. However, the variation of the samples was rather high and values in the MS studies did not reach significance (Table 2). This is very likely since the biopsies contained very variable amounts of epithelial cell layer vs. submucosa.

**Table 2:** Statistical analysis of mass spectrometry after inhalation of a 10% octylglucopyranoside (OGP), a 125 µM or a 500 µM sphingosine (SPH) solution (5 mL). The range of sphingosine (Sph), ceramide 16 (Cer 16), ceramide 18 (Cer 18), ceramide 20 (Cer20), ceramide 22 (Cer 22), ceramide 24 (Cer 24), ceramide 24:1 (Cer 24:1) and ceramide total (Cer total) in pmol/mg protein.

	OGP		125 µM SPH		500 µM SPH		*p*-values
	n	Mean ± sd	n	Mean ± sd	n	mean ± sd	
**Sph**							
Main bronchus	3	68 ± 31	3	180 ± 55	3	107 ± 39	n.s
Bronchus	3	49 ± 14	3	105 ± 31	3	71 ± 56	n.s
Periphery	3	134 ± 14	3	276 ± 192	3	223 ± 27	n.s
**Cer 16**							
Main bronchus	3	696 ± 402	3	718 ± 361	3	571 ± 130	n.s
Bronchus	3	513 ± 349	3	965 ± 664	3	447 ± 159	n.s
Periphery	3	628 ± 196	3	744 ± 276	3	1507 ± 756	n.s
**Cer 18**							
Main bronchus	3	115 ± 74	3	211 ± 188	3	126 ± 33	n.s
Bronchus	3	132 ± 64	3	295 ± 75	3	114 ± 25	n.s
Periphery	3	150 ± 70	3	188 ± 123	3	418 ± 216	n.s
**Cer 20**							
Main bronchus	3	98 ± 79	3	106 ± 52	3	67 ± 11	n.s
Bronchus	3	75 ± 40	3	121 ± 64	3	285 ± 199	n.s
Periphery	3	90 ± 41	3	94 ± 16	3	127 ± 100	n.s
**Cer 22**							
Main bronchus	3	199 ± 140	3	274 ± 141	3	195 ± 26	n.s
Bronchus	3	129 ± 59	3	432 ± 191	3	141 ± 17	n.s
Periphery	3	229 ± 79	3	320 ± 90	3	555 ± 333	n.s
**Cer 24**							
Main bronchus	3	392 ± 271	3	543 ± 146	3	453 ± 165	n.s
Bronchus	3	209 ± 84	3	739 ± 354	3	284 ± 111	n.s
Periphery	3	508 ± 89	3	660 ± 139	3	942 ± 526	n.s
**Cer 24:1**							
Main bronchus	3	334 ± 274	3	533 ± 136	3	479 ± 202	n.s
Bronchus	3	222 ± 101	3	445 ± 170	3	271 ± 178	n.s
Periphery	3	447 ± 194	3	546 ± 38	3	691 ± 238	n.s
**Cer total**							
Main bronchus	3	1834 ± 1211	3	2387 ± 1024	3	1917 ± 339	n.s
Bronchus	3	1278 ± 604	3	3076 ± 1216	3	1322 ± 426	n.s
Periphery	3	2052 ± 342	3	2577 ± 623	3	4398 ± 2210	n.s

Tissue samples from main bronchus, bronchus and periphery were collected after a 4-h EVLP run. Given is the mean ± SD, ANOVA.

**Figure 1:**
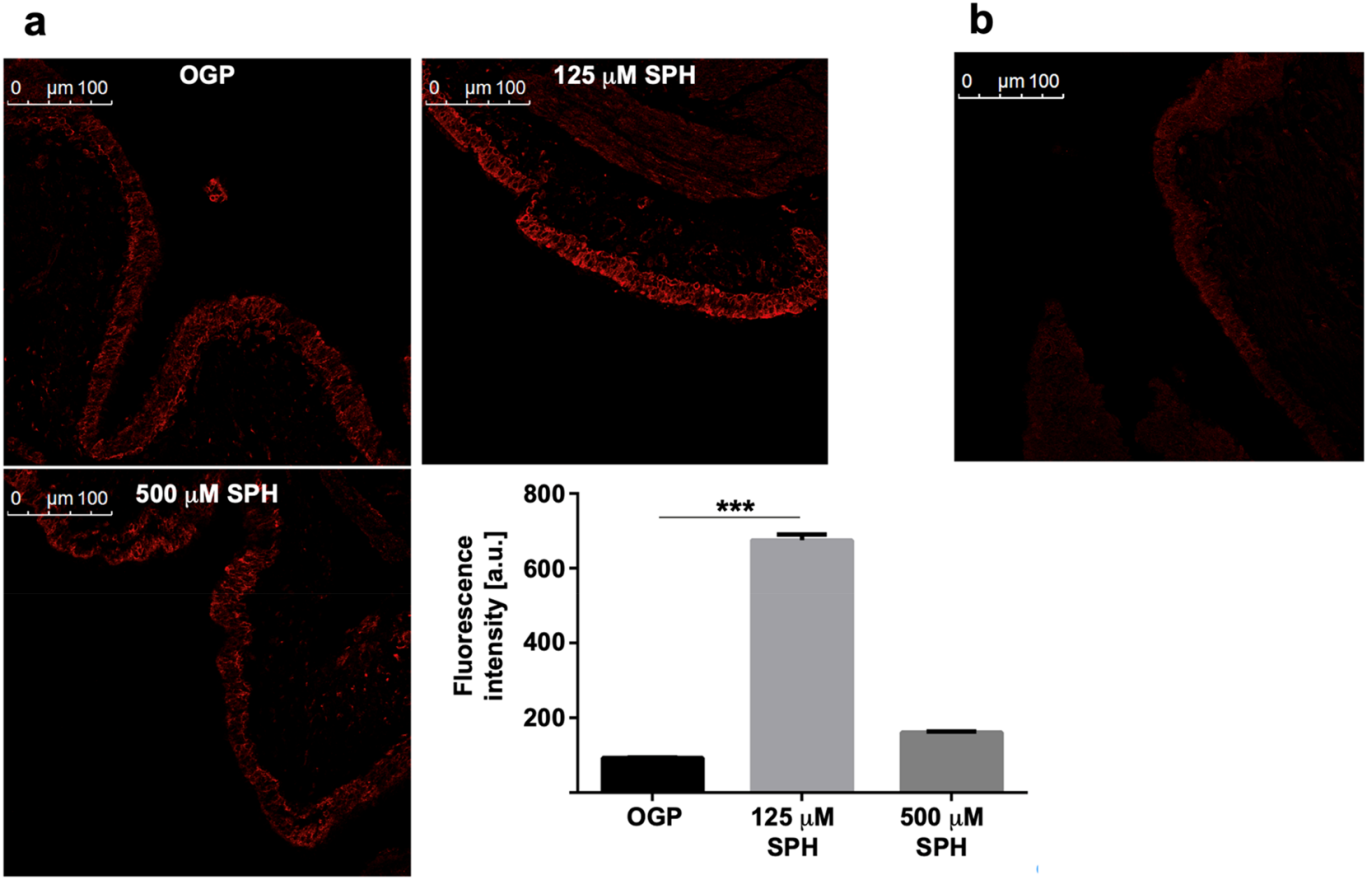
(**a**, **b**) Fluorescence intensity after staining with Cy3-coupled anti-sphingosine (**a**) and autofluorescence intensity without staining as control (**b**). Histological studies demonstrate an accumulation of sphingosine in bronchial epithelial cells after inhalation. EVLP minipig lungs were inhaled with sphingosine at concentrations of 125 µM sphingosine (SPH), 500 µM sphingosine (SPH) and with 0.125% octylglucopyranoside (OGP) as control. The lungs were subjected to bronchoscopy 30 and 150 min after the inhalation, biopsies were fixed in paraformaldehyde, embedded in paraffin and sectioned. Sections were stained with Cy3-coupled anti-sphingosine and without coupling (control). Shown are representative immune stainings. Given is the mean ± SD from 3 sections with 5 visual fields per animal (blinded tests), *p < 0.05, **p < 0.01, ***p < 0.001, ANOVA.

Since MS of biopsies determines sphingosine not only in the epithelial cells that are exposed to sphingosine, but also in the submucosa and other bronchial tissue such as muscles and even small vessels, we analyzed whether inhalation of sphingosine results in an increase of sphingosine specifically in the bronchial epithelial cell (BEC) layer. To this end, we performed histological studies and stained paraffin sections with Cy3-coupled monoclonal anti-sphingosine antibodies. The results show a marked increase of sphingosine specifically in bronchial epithelial cell layers after tube-inhalation of a 125 μM SPH suspension compared to the solvent OGP (Fig. 1a). We did not observe an accumulation of sphingosine in the submucosa or in endothelial cells (Fig. 1a). Interestingly, tube-inhalation with sphingosine suspension at 500 μM did not increase its local concentration in the BEC layer compared to 125 µM (Fig. 1a), which might be caused by the generation of larger micelles that are unable to interact with cells at this high concentration. An autofluorescence intensity without staining was performed as control (Fig. 1b).

**Figure 2:**
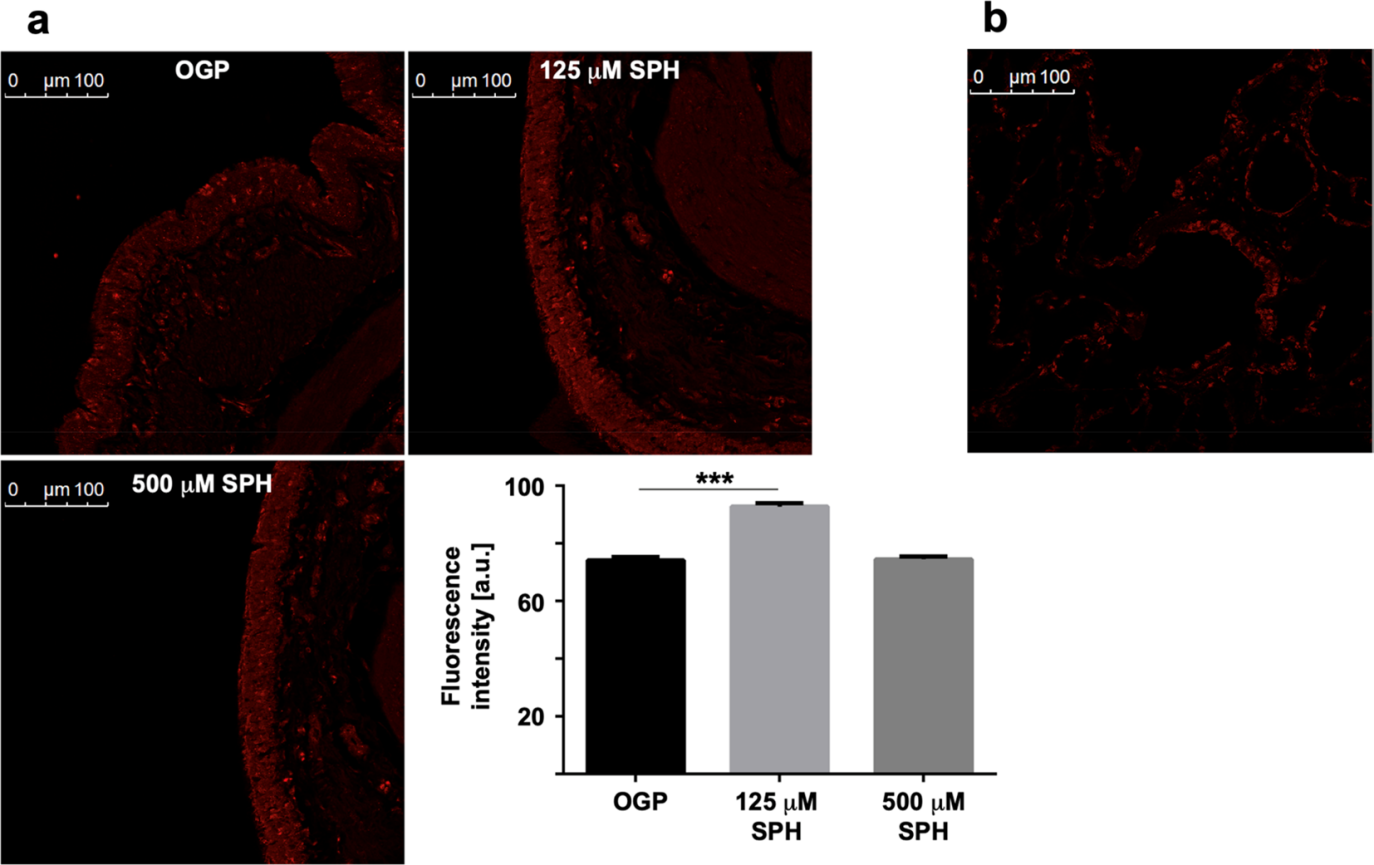
(**a**, **b**) Fluorescence intensity after staining with Cy3-coupled anti-ceramide (**a**) antibodies and autofluorescence intensity without staining as control (**b**). EVLP minipig lungs were inhaled with sphingosine at concentrations of 125 µM sphingosine (SPH), 500 µM sphingosine (SPH) and with 0.125% octylglucopyranoside (OGP) as control. The lungs were subjected to bronchoscopy 30 and 150 min after the inhalation, biopsies were fixed in paraformaldehyde, embedded in paraffin and sectioned. Sections were stained with Cy3-coupled anti-ceramide antibodies and without coupling (control). Shown are representative immune stainings. Given is the mean ± SD from 3 sections with 5 visual fields per animal (blinded tests), *p < 0.05, **p < 0.01, ***p < 0.001, ANOVA.

Next, we tested whether sphingosine is converted into ceramide within BEC after tube-inhalation. Biopsies from lungs after tube-inhalation reveal a small, but significant increase of ceramide concentrations in BEC compared to OGP (Fig. 2a). An autofluorescence intensity without staining was performed as control (Fig. 2b). The MS studies on ceramide did not reach statistical significance, again due to the relatively high variation of the values (Table 2).

**Figure 3:**
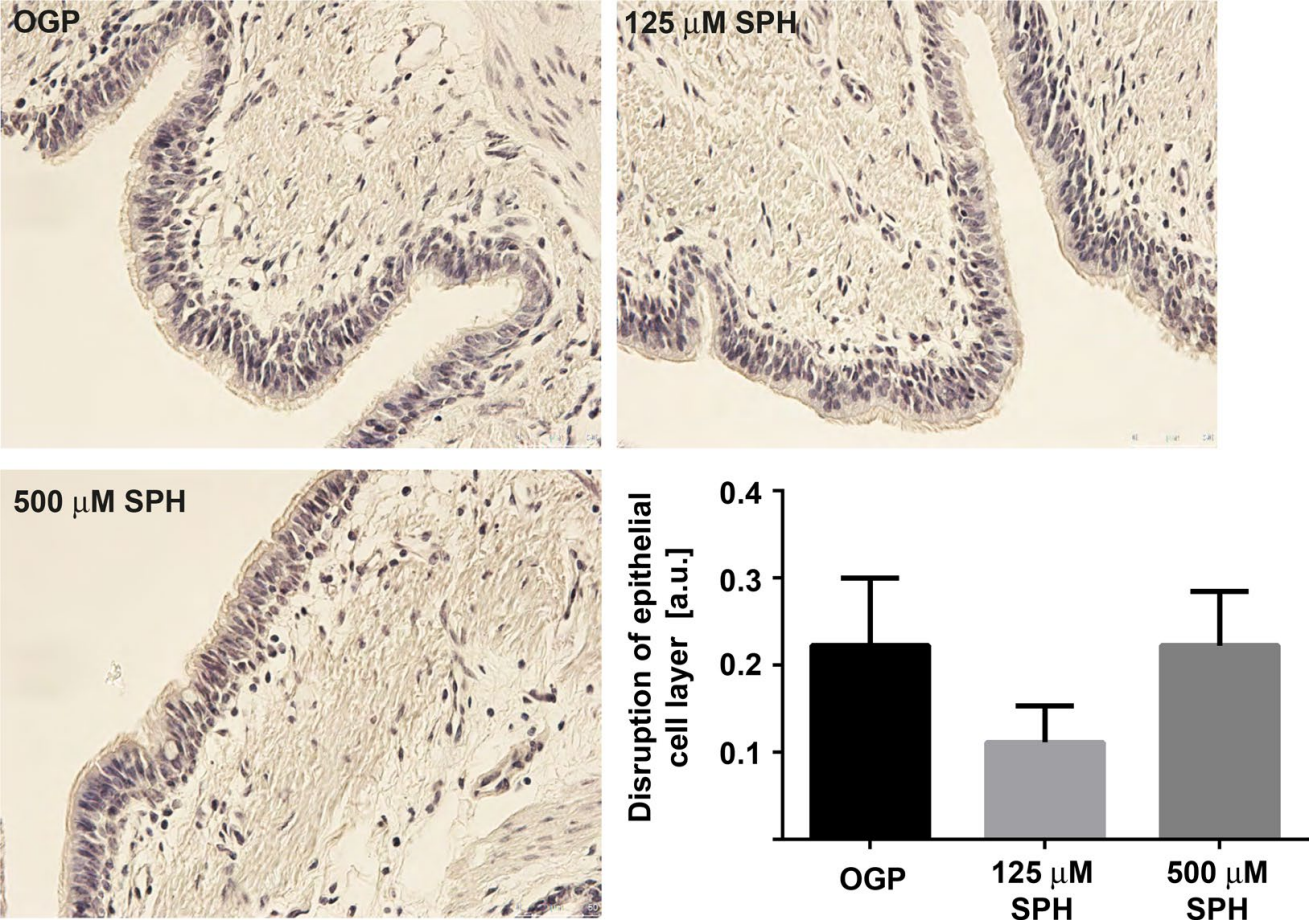
Sphingosine inhalation does not lead to disruption of epithelial layers. No loss of cell integrity after inhalation of sphingosine was observed. Paraffin sections from bronchial epithelial cell layers were stained with hemalaun. Out of each EVLP lung (125 µM sphingosine (n = 3), 500 µM sphingosine (n = 3) and OGP (n = 3) 3 sections with 5 visual fields were analyzed according to the following scoring system: No evidence of epithelial cell layer disruption and intact basal membrane, no leukocyte influx and less than 2% pyknotic, i.e. dead epithelial cells (Grade 0); Only small disruptions of the epithelial cell layer but basal membrane intact and no evidence of leukocyte influx with less than 5% pyknotic, i.e. dead epithelial cells (Grade 1); Larger disruptions of the epithelial cell layer are observed but basal membrane is still intact, no evidence of leukocyte influx and less than 10% pyknotic, i.e. dead epithelial cells were shown (Grade 2): Large disruptions of the epithelial cell layer, disruptions of basal membrane, leukocyte influx and more than 10% pyknotic, i.e. dead epithelial cells (Grade 3). Given are representative stainings and the mean ± SD from 3 sections with 5 visual fields per animal (blinded tests), ANOVA.

Next, we analyzed whether sphingosine inhalation affects epithelial cell integrity. To this end, we analyzed the integrity of the bronchial epithelial cell layers upon H&E staining of paraffin sections. The results demonstrate that sphingosine had no negative impact on the integrity of the BEC layer (Fig. 3).

**Figure 4:**
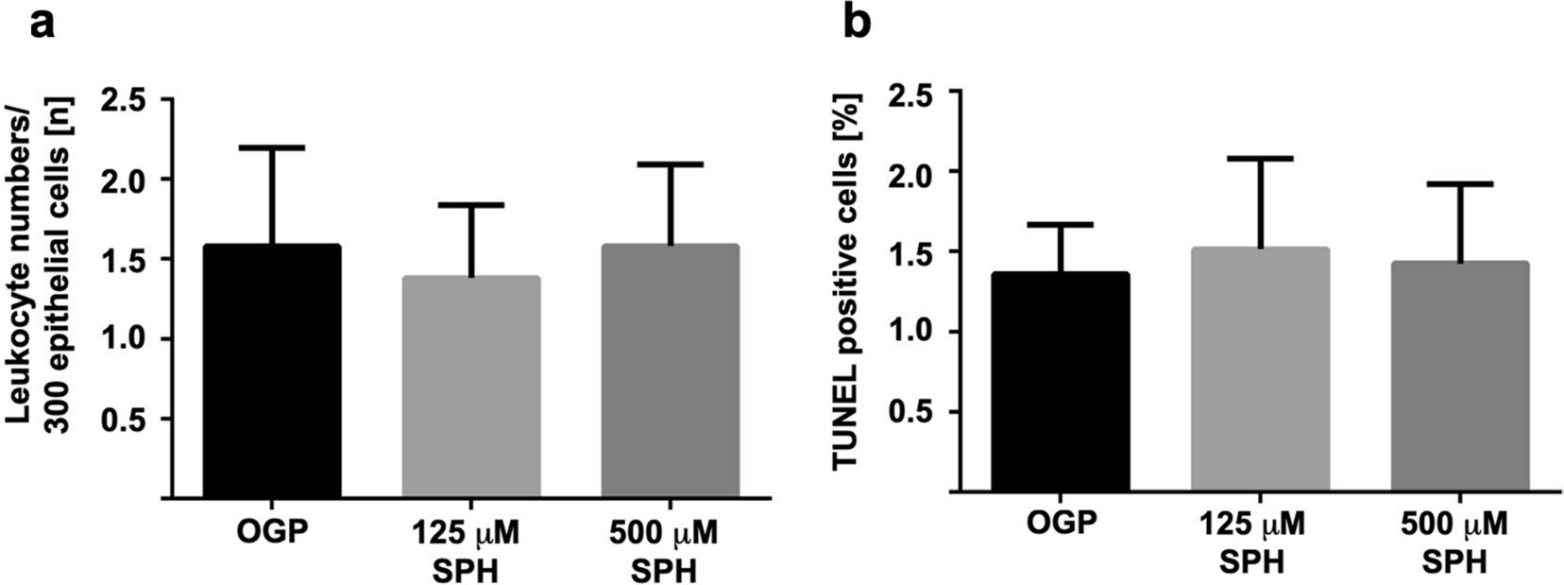
(**a**, **b**) Leukocyte numbers/300 epithelial cells (**a**); TUNEL positive cells (**b**). No higher incidence of cell death or leukocyte influx in bronchial epithelial cells was observed after inhalation of sphingosine. Hemalaun staining was performed for paraffin sections from bronchial biopsies from EVLP minipig lungs that were inhaled with 125 µM sphingosine (SPH), 500 µM sphingosine (SPH) or with octylglucopyranoside (OGP) as control. Three sections with 5 visual fields were analyzed for each lung and leukocyte numbers per 300 epithelial cells were counted. TUNEL reaction was performed for paraffin sections from bronchial biopsies from EVLP minipig lungs that were inhaled with 125 µM sphingosine (SPH), 500 µM sphingosine (SPH) or with octylglucopyranoside (OGP) as control. Statistical analysis of leukocyte influx and TUNEL positive cells after inhalation of octylglucopyranoside (OGP), 125 µM (125 µM SPH) or 500 µM (500 µM SPH) sphingosine containing 5 ml suspension did not reveal a statistically significant difference. Given are numbers of leukocytes/300 epithelial cells (n) and percentage of TUNEL positive cells from 3 sections with 5 visual fields per animal (blinded tests), Given are mean ± SD, ANOVA.

Sphingosine could be converted into sphingosine 1-phosphate, which might induce an influx of leukocytes into the tissue. Thus, we determined the number of leukocytes within the BEC layer upon tube-inhalation. These studies revealed that inhalation of sphingosine did not induce a leukocyte-influx into the BEC layer (Fig. 4). Finally, we also determined whether sphingosine-inhalation might induce cell death in the BEC layer. To this end, we performed TUNEL assays on paraffin sections from bronchi prior and after sphingosine-inhalation. The results showed no evidence for any induction of cell death by sphingosine (Fig. 4).

## Discussion

In the present study, we demonstrate that sphingosine-inhalation in EVLP minipig lungs has no side effects in the trachea and the lung. We also did not detect any effects to the functional performance during a 4-h EVLP run. No increase in lactate levels or activity of LDH and AP in the perfusion perfusate was observed.

The present data are consistent with previous *in-vivo* inhalation studies in mice and mini pigs showing that nasal inhalation of sphingosine has no adverse side effects^28,29^. However, in these studies sphingosine was applied via nasal inhalation, which does not allow application of such a defined dose of sphingosine as in the present study. Further, previous studies did not record any functional lung data. In our previous study healthy mini pigs underwent a 14-day period of sphingosine inhalation. It was shown that the daily administration did not result in obvious changes of the health status, loss of activity or reduced food intake and no local signs of inflammation in the upper airway were observed, consistent with the present data.

To measure the sphingosine concentration specifically in epithelial cells of the bronchi and trachea we performed confocal microscopy. These studies revealed an increase of the surface sphingosine concentration in the bronchial epithelial cell layer in EVLP minipig lungs upon application of sphingosine. Likewise, the mass spectrometry studies showed increasing concentrations of sphingosine after inhalation without reaching statistical significance, which is likely due to the low number of samples. It is important to note that we detected only a very small increase of ceramide in the epithelial cell layer of the bronchi after application of 125 µM sphingosine and a slightly more pronounced increase after administration of 500 µM sphingosine. This might be due to a very low conversion of sphingosine into ceramide or by a rapid conversion of ceramide into other lipids. We also did not detect an increase of sphingosine 1-phosphate in biopsies of bronchi upon inhalation suggesting that this metabolite of sphingosine is either not formed or also rapidly consumed. However, it is also possible that the concentrations of sphingosine 1-phosphate in the biopsies were below the detection level. In any case, the concentrations of sphingosine 1-phosphate are very low, which is important since increased concentrations of sphingosine 1-phosphate might trigger an influx of leukocytes into the bronchi. An influx of neutrophils cannot be determined in the present system, which is an isolated perfused system.

It is also interesting to note that we did not observe a linear increase of sphingosine concentrations in the BEC layer with increased concentration of sphingosine in the inhalation fluid. We have already observed a similar phenomenon in the in vivo studies on minipigs with nasal inhalation of sphingosine^28^. It might be possible that higher concentrations of sphingosine form larger micelles that are less efficient aerosolized or too large to be carried for a longer distance in the airways. Thus, a dose of 125 μM sphingosine in the inhalation fluid seems to be optimal.

Sphingosine is a long chain base that is part of the lipid composition of the bronchial epithelial cell layer and inter alia important for the first line defense against pathogens. Low sphingosine levels were detected in the respiratory tract in patients and mice with cystic fibrosis as well as in mice after burn injury or in elderly mice. All of these mice showed an increased susceptibility for pulmonary infections, which was corrected by inhalation of sphingosine^12,30–32^, indicating the significance of sphingosine for the defense of the airways against pulmonary infections. Studies in recent years have shown that an increase of sphingosine levels via the sphingomyelin-pathway or upon direct administration of exogenous sphingosine by inhalation or tube-coating leads to decreased susceptibility of pulmonary infection, significant reduction in colony forming units (CFU) in infected mice as well as decreased mortality rates in these mice models^8,12,29,31,33–37^.

The present study supports the notion that sphingosine might serve as a new therapeutic treatment option with no side effects and a broad-spectrum antibacterial activity^8,29–31,33^. Further research is required to investigate the antibacterial effects of inhaled sphingosine in EVLP lungs. Strength of the model system was a controlled and precise application of therapeutics as well as the opportunity to examine lung performance, macroscopic changes and the implementation of a broncoscopy during the EVLP run. Limitations are more or less pronounced damages in lung parenchyma due to ventilation and perfusion which could induce barotrauma or congestion with its deleterious effects.

## Conclusion

In summary, we demonstrate that inhalation of sphingosine into an EVLP minipig lung results in an increase of sphingosine concentrations in bronchial epithelial cells. The inhalation has neither local side effects nor affects functional parameters during the 4 h EVLP run.
